# Draft genome and antibiotic resistance profile of high-risk ST15 multidrug-resistant *Klebsiella pneumoniae* strain Kleb009 from a neonatal blood culture in Ghana

**DOI:** 10.1128/mra.01202-25

**Published:** 2026-01-23

**Authors:** Francis Kwame Morgan Tetteh, Kashif Haq, William Boateng, Kenechukwu Egwuonwu, Beverly Egyir, Eric Sampane-Donkor, Kwabena Obeng Duedu

**Affiliations:** 1 Department of Medical Microbiology, University of Ghana Medical School 63533, Accra, Ghana; 2 Pathology Division, 37 Military Hospital, Accra, Ghana; 3 Department of Life and Sport Sciences, School of Life and Health Sciences, Birmingham City University 1725, Birmingham, United Kingdom; 4 Department of Bacteriology, Noguchi Memorial Institute for Medical Research, College of Health Sciences, University of Ghana 58835, Accra, Ghana; 5 Allison M. Howell Centre for Religion, Environment, Science and Development, Akrofi Christaller Institute of Theology, Mission and Culture 125561, Akropong-Akuapem, Ghana; University of Maryland Baltimore, Baltimore, Maryland, USA

**Keywords:** *Klebsiella pneumoniae*, antibiotic resistance, genome analysis, genomes

## Abstract

We present the draft genome sequence and antibiogram of a high-risk sequence type ST15 multidrug-resistant *Klebsiella pneumoniae* strain (Kleb009) from a neonatal blood culture from Ghana. Despite the public health importance of *K. pneumoniae*, genomic data on locally circulating strains are limited. This data will enhance antimicrobial resistance surveillance efforts.

## ANNOUNCEMENT

*Klebsiella pneumoniae* is ranked the second most common cause of gram-negative bacteremia worldwide and the leading cause of neonatal sepsis in sub-Saharan Africa (SSA), with over 600,000 deaths attributed to drug-resistant *K. pneumoniae* strains in the region in 2019 (1, 2). The involvement of multidrug-resistant (MDR) ST15 *K. pneumoniae* in neonatal bloodstream infections has been less frequently reported in SSA including Ghana. Understanding the genomic landscape of MDR *K. pneumoniae*, particularly high-risk clones such as ST15, can provide insights into resistance mechanisms, transmission dynamics, and potential therapeutic targets. Here, we report the draft genome sequence and antibiogram profile of a high-risk MDR *K. pneumoniae* strain (Kleb009) recovered from the blood culture of a day-old neonate with sepsis during a cross-sectional study in Southern Ghana between April and December 2021.

Briefly, the skimmed-milk tryptone glucose glycerol stock of the *K. pneumoniae* was inoculated into tryptic soy broth (Oxoid, UK) and incubated at 37°C for 24 h. The culture was sub-culturedonto nutrient agar (Oxoid) and incubated at 37°C for 20 h. Species identity was confirmed using a BD MALDI-TOF analyzer version 4.2.100 analyzer (BRUKER Daltonics, Germany). Antibiotic susceptibility testing was performed using MicroScan autoSCAN-4 system (American MicroScan, USA) according to the manufacturer’s instructions and interpreted using CLSI M100 guidelines (3). The isolate was inoculated into microdilution panels containing preloaded antibiotics, incubated at 37°C for 20 h, and scanned for absorbance representing growth. *K. pneumoniae* NCTC 13438 and *Escherichia coli* ATCC 25922 were used as the quality control strains for the minimum inhibition concentration test (see Table S1 at https://doi.org/10.5281/zenodo.16878187).

Genomic DNA was extracted from overnight nutrient broth cultures at 37°C using the QIAamp DNA Mini Kit (Qiagen GmbH, Germany). The isolate had a DNA concentration ≥10 ng/μL as measured with the Qubit 4.0 Fluorometer (Thermo Fisher Scientific, USA), and was used for library preparation with the Illumina DNA Prep (M) Tagmentation Kit (Illumina Inc., USA). By following the manufacturer’s manual, libraries underwent enzymatic fragmentation, adapter tagmentation, limited-cycle PCR with dual indexing, and size selection. Fragment sizes were assessed using the Agilent 2100 Bioanalyzer (Agilent Technologies, USA), re-quantified with Qubit 4.0 Fluorometer, normalized to 2 nM, and sequenced on the Illumina NextSeq 1000 using paired-end (2 × 150 bp) chemistry. Trim Galore v0.6.10 (https://github.com/FelixKrueger/TrimGalore?tab=readme-ov-file) and Spades v4.2.0 (4) were selected for read quality control and adapter trimming, and *de novo* assembly respectively in the Genome Assembly tool from the BV-BRC v3.54.6a platform (https://www.bv-brc.org/). Default genomic metrics including target genome coverage of 200, minimum contig length of 300, and minimum contig coverage of 5 were used. Assembly data is presented in Table 1. Genome annotation was performed using PGAP v6.10 (5). Kleborate v3.2.4 (6) was used for speciation, determination of resistance and virulence genes phenotype, and multi locus sequence type. PlasmidFinder v2.1 (7) was used in determining plasmid replicons. All analyses were performed using default parameters except where mentioned.

**TABLE 1:** Genome assembly profile of *K. pneumoniae* strain *Kleb009^a^*

Feature	Result or value
Sample ID	Francis_KA009
Strain ID	Kleb009
No. of contigs	139
GC content (%)	56.98
Contig *L*_50_	14
Genome length (bp)	5,666,003
Contig *N*_50_ (bp)	138,810
Coverage	325
No. of reads	130,574,204
No. of total genes	5,566
No. of total coding sequences (CDS)	5,484
No. of tRNAs	65
No. of rRNAs	7

^*a*^ Outputs from the BV-BRC Genome Assembly tool. *Klebsiella pneumoniae* strain Kleb009 was assembled using SPAdes v4.2.0 (4). The *N*_50_ length is defined as the shortest sequence length at 50% of the genome and the *L*_50_ count is defined as the smallest number of contigs whose length sum produces *N*_50_. The genome was annotated using PGAP v6.10 (5).

## Data Availability

Assembled data were deposited in the National Center for Biotechnology Information (NCBI) database under the BioProject accession number PRJNA1265775. The BioSample accession number is SAMN50501738. The assembly accession number is GCA_052828425.1, and the WGS project accession number is JBQRKR000000000.
